# Dynamic Compressive Behavior and Deformation-Failure Characteristics of a Medium-Carbon Cr-Ni-Mo High-Strength Steel

**DOI:** 10.3390/ma19204302

**Published:** 2026-10-13

**Authors:** Yi Qu, Ao Zhang, Ronghua Su, Bo Yang, Zhenlong Chao, Yunhou Sun

**Affiliations:** 1 Defense Engineering Institute, AMS, PLA, Beijing 100850, China; 2 College of Aerospace and Civil Engineering, Harbin Engineering University, Harbin 150001, China; 3 School of Materials Science and Engineering, Harbin Institute of Technology, Harbin 150001, China

**Keywords:** medium-carbon Cr-Ni-Mo high-strength steel, dynamic compressive behavior, strain-rate and temperature effects, Johnson–Cook model, deformation and failure characteristics

## Abstract

**Highlights:**

- Medium-carbon Cr-Ni-Mo high-strength steel exhibits pronounced strain-rate strengthening and thermal softening, with a mean maximum compressive stress of 2500.9 MPa at 298 K and 5000 s^−1^.
- Increasing strain rate promotes inclined shear cracking and enhances shear-related fracture features; the calibrated Johnson–Cook model reasonably reproduces the stress response and major deformation and failure characteristics.
- The combined experimental, microscopic, and numerical results provide a more complete characterization of the dynamic deformation and damage behavior of medium-carbon Cr-Ni-Mo high-strength steel.
- The calibrated constitutive and damage parameters provide reference data for numerical simulation and dynamic performance assessment of high-strength steels.

**Abstract:**

To investigate the dynamic compressive behavior and deformation characteristics of medium-carbon Cr-Ni-Mo high-strength steel under different strain rates and temperatures, quasi-static tensile and compression tests, together with Split Hopkinson Pressure Bar (SHPB) dynamic compression tests, were conducted. The Johnson–Cook flow stress and damage model parameters were calibrated based on the experimental results under the investigated loading conditions. Combined with macroscopic morphologies of recovered specimens, SEM fracture observations, and finite element simulations, the deformation and damage behavior of the material was investigated. The results show that the material exhibits pronounced strain-rate strengthening and thermal softening characteristics. At 298 K, increasing the strain rate from 1000 to 5000 s^−1^ increases the mean maximum compressive stress from 2263.1 to 2500.9 MPa, while expanding the plastic deformation range. At 1000 s^−1^, increasing the temperature from 298 to 698 K decreases the mean maximum compressive stress from 2263.1 to 1722.2 MPa. The calibrated Johnson–Cook model reasonably describes the stress response and deformation characteristics at different strain rates at 298 K, with relative errors of simulated maximum compressive stresses below 4% for the representative experimental curves used in the numerical comparison. The macroscopic and microscopic observations reveal that increasing strain rate promotes the localized shear deformation, inclined cracking, and partial fragmentation after plastic compression, with enhanced shear deformation features reflected by elongated dimples, fracture steps, and tear ridges. Increasing temperature reduces fragmentation under high strain-rate loading and promotes the formation of coarse dimples and plastic flow features. Numerical analysis further indicates that increasing strain rate promotes the localization of equivalent plastic strain, leading to inclined shear bands and crack formation.

## 1. Introduction

With the development of aerospace, rail transportation, energy equipment, and marine engineering, the service environments and loading conditions of critical load-bearing components have become increasingly complex, imposing higher requirements on the strength, toughness, and dynamic mechanical properties of materials [1,2]. High-strength steels and ultra-high-strength steels possess excellent mechanical properties and a favorable strength-toughness balance, and are therefore widely used in high-load-bearing components [3,4]. Among them, Cr-Ni-Mo-based high-strength steels such as 300 M, 18CrNiMo7-6, 34CrNi3MoA, and AISI 4330 have been employed in aircraft landing gears, high-speed train and wind turbine gears, heavy-duty power equipment, and oil and gas equipment [4,5,6,7,8]. During service, these components are subjected not only to static and cyclic loads but also to transient loads such as impact and vibration, under which the materials exhibit dynamic mechanical responses and failure characteristics distinct from those under quasi-static conditions. Therefore, investigating the mechanical response and deformation-failure behavior of high-strength steels under high strain rates is of great significance for understanding their dynamic properties and failure mechanisms [9,10].

To date, extensive studies have been conducted on the mechanical responses of high-strength steels under high strain-rate conditions. Fan [11] found, through quasi-static compression and SHPB dynamic compression tests, that the flow stress of 22SiMn2TiB high-strength steel increased from approximately 1600 MPa under quasi-static loading to approximately 2400 MPa at 5000 s^−1^, indicating a pronounced strain-rate strengthening effect. Wei [12] similarly reported for Q690D QT high-strength steel that the flow stress increased markedly with increasing strain rate, whereas an increase in temperature reduced its flow stress. In recent years, related studies have further focused on the combined effects of temperature, strain rate, and microstructural state on the dynamic mechanical response. Gronostajski [13] investigated the high-strain-rate deformation behavior of TRIP690 and DP600 advanced high-strength steels and found that the adiabatic temperature rise induced by rapid plastic deformation significantly affected the flow response and microstructural evolution, particularly the transformation behavior of retained austenite. Oliveira [14] conducted Hopkinson pressure bar dynamic compression tests on high-hardness steel with different tempered microstructures and found that the initial microstructural state strongly affected the susceptibility to adiabatic shear localization, while significant grain refinement and dynamic recrystallization occurred within the transformed shear bands. Zhong [15] compared the dynamic behavior of 18CrNiMo7-6 steel under different heat treatment conditions, and the results showed that the dynamic stress level and temperature-sensitive range differed markedly among the normalized, quenched, and carburized-quenched states. Guan [16] further examined the dynamic compression behavior of 34CrNi3MoA steel over the ranges of 25–600 °C and 1000–4600 s^−1^, and found that both the strain-rate sensitivity and strain-hardening behavior varied with temperature and plastic strain. Overall, recent dynamic mechanical studies of high-strength steels have gradually evolved from investigating isolated strain-rate strengthening effects toward understanding flow behavior and microstructural evolution under the coupled effects of temperature, strain rate, and initial microstructure.

In addition to variations in dynamic strength, high strain-rate loading also alters the deformation and failure modes of high-strength steels. Baik [17] investigated the thermoplastic microstructural evolution of adiabatic shear bands in a high-strength and high-toughness 10 wt.% Ni steel and found that severe strain localization was accompanied by pronounced microstructural refinement within the shear bands, including lattice rotation, subdivision of the original microstructure, and the formation of ultrafine recrystallized grains. Jin [18] observed a similar evolution process in a 2000 MPa-grade ultra-high-strength martensitic steel: with increasing deformation, deformed shear bands gradually developed into transformed shear bands, and cracks subsequently initiated and propagated within the shear bands, ultimately leading to material failure. Zhou [19] also found in a medium-carbon bainitic steel that dislocation multiplication, lath fragmentation, and subgrain formation occurred within the material under high strain rates, and pronounced adiabatic shear localization appeared at even higher strain rates. From the perspective of fracture morphology, Roth and Mohr [20] demonstrated that the fracture response of advanced high-strength steels was strongly rate-dependent, with the strain to fracture varying markedly with loading rate, indicating that dynamic loading can significantly modify the initiation and development of ductile fracture. These studies show that the dynamic failure of high-strength steels is manifested not only by macroscopic shear localization and cracking but also by microstructural subdivision and refinement, dislocation evolution, crack initiation and propagation, and localized plastic deformation. The specific failure mode is therefore closely related to the initial microstructure and loading conditions.

In summary, although the dynamic mechanical behavior and failure characteristics of different high-strength steels have been extensively studied, the dynamic deformation and failure behavior of a specific steel requires further investigation with consideration of its chemical composition, heat-treatment condition, and strength–toughness balance. On this basis, the present study investigates the dynamic compressive behavior of a medium-carbon Cr-Ni-Mo high-strength steel through quasi-static mechanical tests and SHPB dynamic compression tests at different strain rates and temperatures. The strain-rate strengthening, thermal softening, and plastic deformation behavior of the material are systematically analyzed. The experimental results are used to calibrate the Johnson–Cook flow stress and damage parameters, and finite element simulations are performed to examine deformation and damage evolution during dynamic compression. By relating the measured stress–strain responses to the macroscopic morphologies of the recovered specimens and SEM fracture features, this study examines the effects of strain rate and temperature on the compressive deformation and failure behavior of the steel under the investigated heat-treatment condition.

## 2. Materials and Methods

### 2.1. Material and Heat Treatment

A medium-carbon Cr-Ni-Mo high-strength steel was investigated in this study, and its chemical composition is listed in Table 1. The steel was austenitized at 860 °C for 1 h, followed by oil quenching to room temperature. Subsequently, it was tempered at 220 °C for 3 h and air-cooled to room temperature. The average Rockwell hardness of the heat-treated steel was 53.8 HRC. To minimize the influence of variations in material batch and heat-treatment condition on the experimental results, the specimens for quasi-static tensile and compression tests, as well as those for dynamic compression tests, were all machined from heat-treated steel plates obtained from the same material batch.

### 2.2. Quasi-Static Tensile and Compression Tests

**Figure 1:**
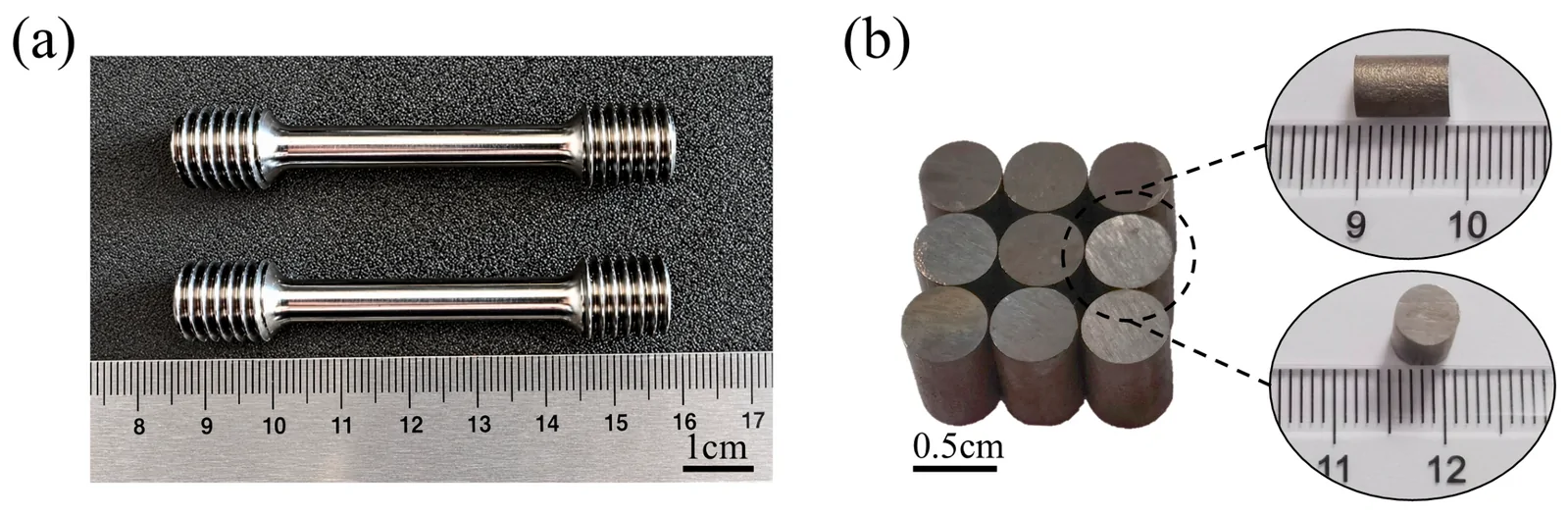
Morphologies of specimens for quasi-static tensile and compression tests: (**a**) quasi-static tensile specimen; (**b**) quasi-static compression specimen.

The quasi-static tensile tests were conducted according to GB/T 228.1–2021 [21]. Specimen deformation was measured using an extensometer with class 1 accuracy. The tensile specimens were cylindrical threaded-end specimens, as shown in Figure 1a, with a gauge-section diameter of 6 mm and an initial gauge length of 20 mm. To ensure the reliability and repeatability of the experimental results, at least three replicate specimens were tested for each testing condition.

The quasi-static compression tests were conducted according to GB/T 7314–2017 [22]. Cylindrical specimens were used, as shown in Figure 1b, with dimensions of Ø5 mm × 8 mm. The crosshead displacement rate was set to 0.5 mm/min, and at least three replicate specimens were tested for each testing condition.

### 2.3. SHPB Dynamic Compression Tests

**Figure 2:**
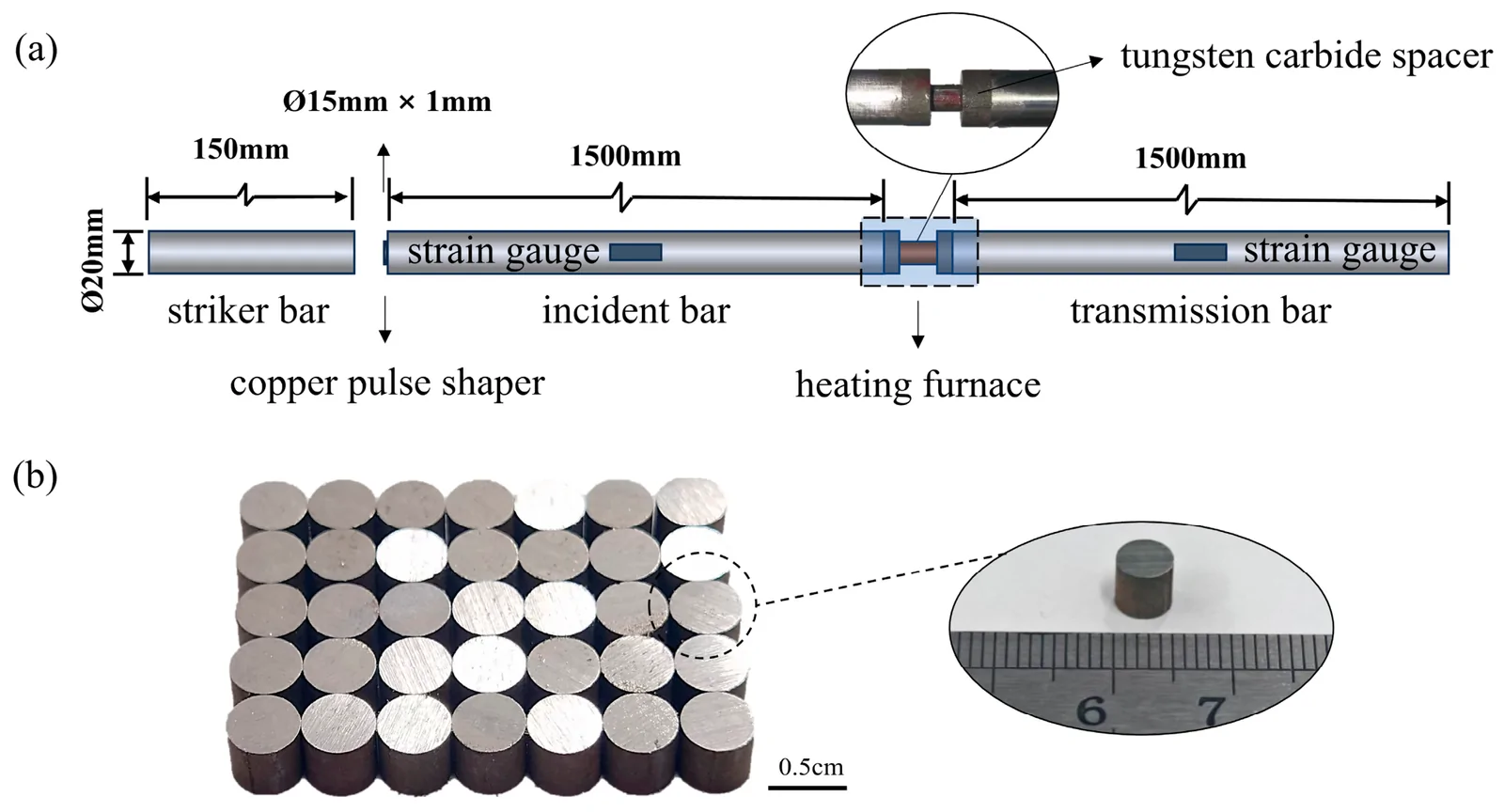
Split Hopkinson Pressure Bar test system and dynamic compression specimens: (**a**) SHPB test system; (**b**) specimens.

The dynamic compression tests were conducted using a Split Hopkinson pressure bar (SHPB) apparatus (Beijing Dongfang Dexing Technology Co., Ltd., Beijing, China), which mainly consisted of a striker bar, an incident bar, and a transmitted bar, as shown in Figure 2a. All three bars were made of high-strength alloy steel and had a diameter of 20.0 mm. The striker bar was 150 mm in length, while the incident and transmitted bars were both 1500 mm long [23]. A pure copper pulse shaper with a diameter of 15 mm and a thickness of 1 mm was used. The dynamic compression specimens were cylindrical, as shown in Figure 2b, with dimensions of Ø5 mm × 5 mm. The specimens were fabricated by wire electrical discharge machining (WEDM), and their end surfaces were sequentially ground using 400–2000 grit abrasive papers to remove machining marks and ensure flat and parallel end faces. Lubricant was applied to the specimen end faces before testing to reduce interfacial friction. At least three replicate specimens were tested for each testing condition.

The test temperatures and strain rates were selected by considering both the requirements of Johnson–Cook model calibration and the testing ranges commonly reported in previous studies on the dynamic compression behavior of high-strength steels. The test temperatures were set to 298, 498, and 698 K, while the target strain rates were 1000, 3000, and 5000 s^−1^, respectively. For the elevated-temperature dynamic compression tests, the specimen temperature was controlled using electromagnetic induction heating [24,25].

### 2.4. Fracture and Deformation Morphology Characterization

After the quasi-static and dynamic compression tests, representative cracked regions and recovered specimens were selected for microscopic characterization. A scanning electron microscope (SEM) (ZEISS, Carl Zeiss Microscopy GmbH, Jena, Germany) was used to examine the crack features, fracture morphologies, and features associated with localized plastic deformation. The SEM observations were performed at an accelerating voltage of 20.0 kV.

## 3. Results

### 3.1. Quasi-Static Mechanical Behavior

**Figure 3:**
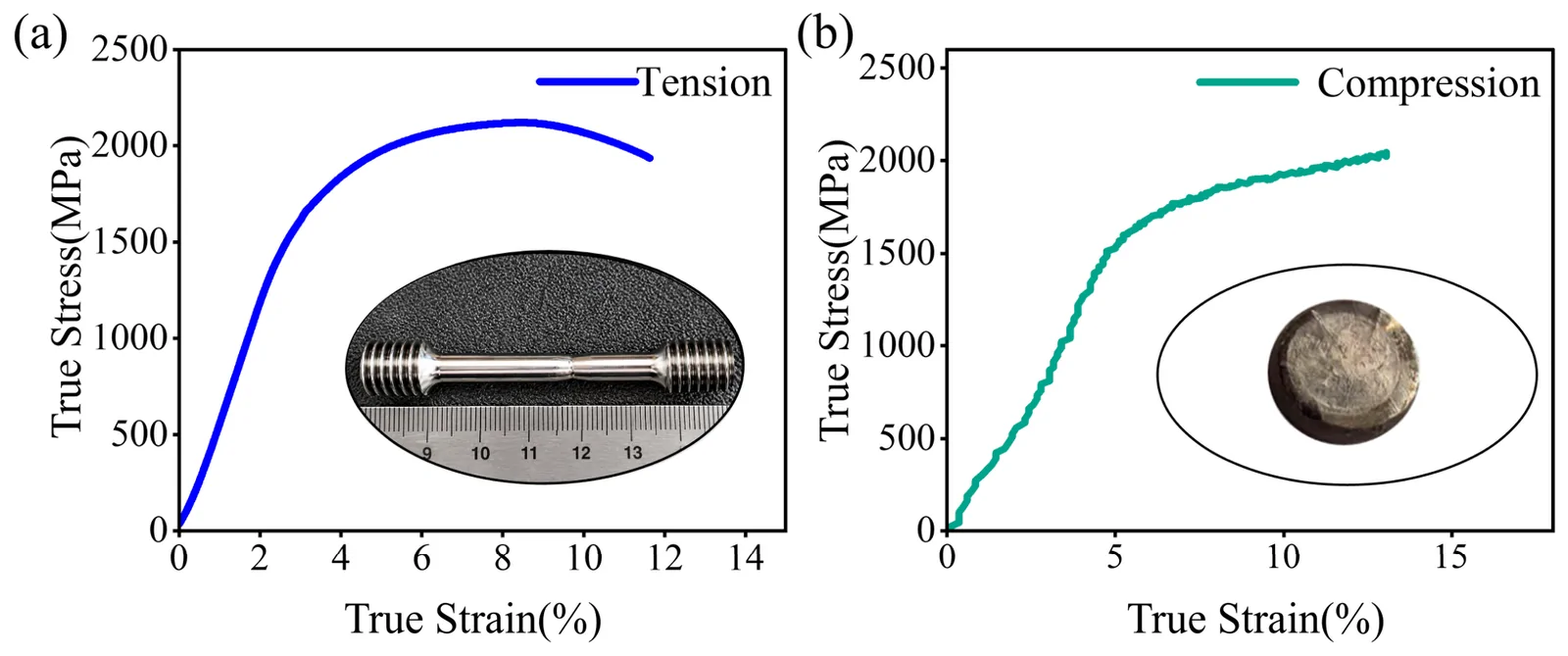
Representative quasi-static true stress–strain curves and post-test specimen morphologies of the medium-carbon Cr-Ni-Mo high-strength steel: (**a**) tension; (**b**) compression.

Figure 3 presents representative true stress–strain curves of the medium-carbon Cr-Ni-Mo high-strength steel under quasi-static tensile and compressive loading, together with the corresponding post-test specimen morphologies. As shown in Figure 3a, the stress increased rapidly with strain during the initial stage of tensile loading. After entering the plastic deformation stage, the material exhibited a pronounced strain-hardening response, with the stress continuing to increase and reaching approximately 2119.6 MPa at a strain of about 0.08 before gradually decreasing. The mean peak stress obtained from three repeated tensile tests was 2113.3 ± 79.77 MPa (mean ± standard deviation, n = 3). Examination of the post-test specimen revealed pronounced localized necking. As shown in Figure 3b, under quasi-static compression, the stress initially increased approximately linearly with strain before the specimen entered the plastic deformation stage. Within the investigated strain range, the stress continued to increase with increasing strain, reaching 2044.8 MPa at a strain of approximately 0.13. The mean maximum compressive stress obtained from the repeated compression tests was 1988.1 ± 65.4 MPa (mean ± standard deviation, n = 3). After testing, the specimen exhibited distinct barrel-shaped upsetting and an inclined shear crack on its lateral surface, but did not separate completely. These observations indicate that the specimen underwent plastic deformation accompanied by local shear cracking under quasi-static compression.

### 3.2. Dynamic Compressive Properties

**Figure 4:**
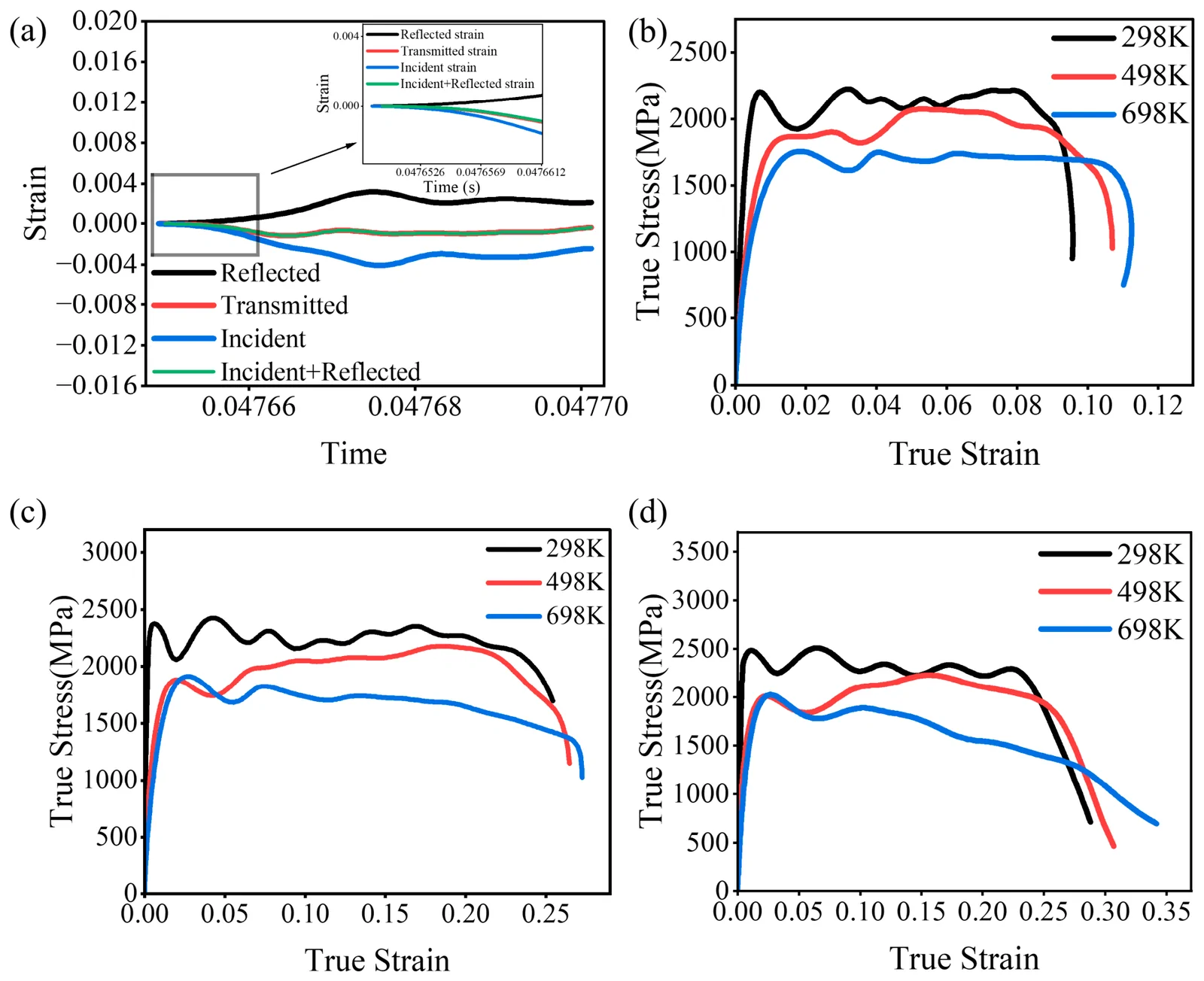
Dynamic compressive responses of the medium-carbon Cr-Ni-Mo high-strength steel at different strain rates and temperatures: (**a**) strain pulses and dynamic stress equilibrium verification at 298 K and 5000 s^−1^; (**b**–**d**) representative true stress–strain curves at different temperatures at strain rates of 1000, 3000, 5000 s^−1^, respectively.

Table 2 summarizes the mean measured strain rates and the mean values and standard deviations of the maximum compressive stress and maximum recorded strain obtained from repeated SHPB tests at each temperature and nominal strain rate. For each test, the measured strain rate was obtained by averaging the experimental strain-rate history over the relatively stable loading stage. For each condition, the mean measured strain rate differed from the nominal value by less than 1%, indicating close agreement with the intended loading rate. Figure 4 presents the dynamic compressive responses of the medium-carbon Cr-Ni-Mo high-strength steel at different temperatures and strain rates. Dynamic stress equilibrium was verified under all nine temperature–strain-rate combinations. A representative comparison between the incident and reflected pulses and the transmitted pulse at 298 K and 5000 s^−1^ is shown in Figure 4a. Good agreement between the two sides of the specimen was observed during the main loading stage, indicating approximate force balance during the SHPB tests [26]. As shown in Figure 4b–d, the representative true stress–strain curves obtained under different strain-rate and temperature conditions exhibit distinct stage-dependent characteristics, which can generally be divided into a rapid stress-rise stage, a plastic-flow stage, and a subsequent stress-drop stage. After yielding, the flow stress remains at a relatively high level over a certain strain range, forming a plateau with stress fluctuations, followed by a rapid decrease at the later stage of loading. Overall, within the dynamic loading range investigated in this study, strain rate and temperature mainly affect the stress level at each deformation stage and the extent of the plastic-flow plateau, whereas the overall evolution of the stress–strain response remains broadly consistent among the different loading conditions.

As shown by the representative stress–strain curves in Figure 4b–d, comparison with the quasi-static compression results reveals a clear strain-rate strengthening effect. Taking 298 K as an example, the overall stress level increases markedly with increasing strain rate. According to the repeated-test results, the mean maximum compressive stress increases from 1988.1 ± 65.4 MPa under quasi-static compression to 2500.9 ± 76.7 MPa at 5000 s^−1^, corresponding to an increase of approximately 25.8%. This result indicates that the medium-carbon Cr-Ni-Mo high-strength steel exhibits pronounced strain-rate sensitivity. Meanwhile, under dynamic loading, the stress–strain curves exhibit a post-yield stress plateau with stress fluctuations. As the strain rate increases, the strain range corresponding to this plateau gradually expands, from approximately 0.01–0.10 at 1000 s^−1^ to approximately 0.03–0.25 at 5000 s^−1^, accompanied by an increase in the maximum recorded strain. These results indicate that, under the tested loading conditions, the specimens sustain relatively high flow stresses over a larger recorded strain range at higher strain rates.

As shown in Figure 4b–d, at a given strain rate, the overall stress level of the material gradually decreases with increasing temperature. Taking 1000 s^−1^ as an example, as the temperature increases from 298 to 698 K, the mean maximum compressive stress decreases from 2263.1 ± 45.9 to 1722.2 ± 34.6 MPa, corresponding to a reduction of approximately 23.9%. This result indicates that the medium-carbon Cr-Ni-Mo high-strength steel exhibits a pronounced thermal softening effect. Meanwhile, with increasing temperature, the strain range corresponding to the post-yield stress plateau expands slightly. The mean maximum recorded strain increases from 0.092 ± 0.004 to 0.113 ± 0.006, corresponding to an increase of approximately 22.8%. Overall, increasing temperature markedly reduces the compressive stress level, while the recorded strain range shows a comparatively modest increase.

### 3.3. Morphology of Specimens Recovered After Dynamic Compression

**Figure 5:**
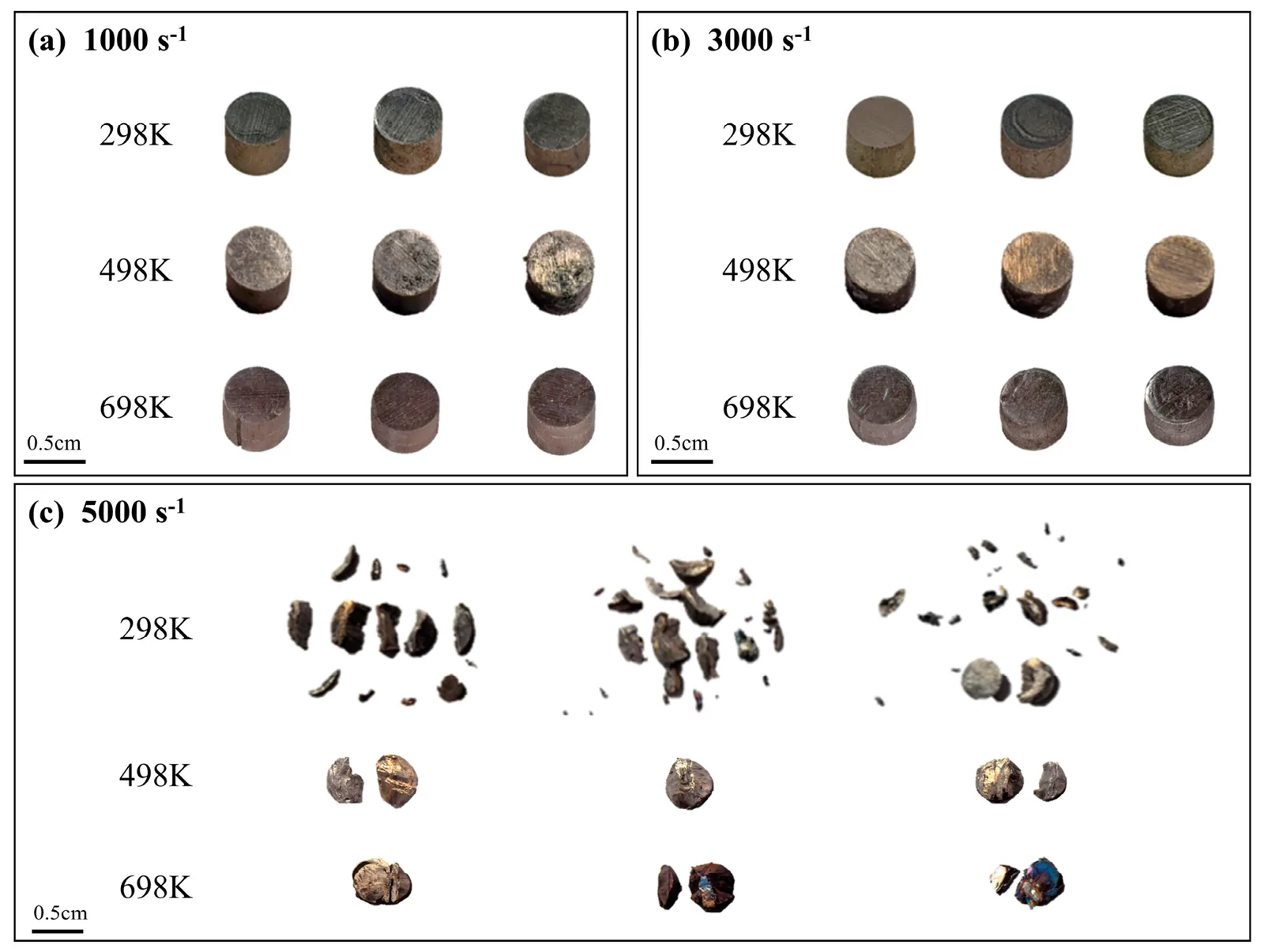
Macroscopic morphologies of specimens recovered after dynamic compression at different temperatures and strain rates: (**a**) 1000 s^−1^; (**b**) 3000 s^−1^; (**c**) 5000 s^−1^.

Figure 5 shows the macroscopic morphologies of the specimens recovered from the repeated tests at different strain rates and temperatures. At 1000 s^−1^, the recovered specimens remain largely intact and mainly exhibit axial shortening accompanied by radial expansion. When the strain rate increases to 3000 s^−1^, inclined cracks appear on the surfaces of some specimens, although the specimens remain largely intact. This indicates that plastic compressive deformation remains dominant under this condition, accompanied by a certain degree of localized shear damage. Consistently, the corresponding stress–strain curves maintain a distinct plastic-flow plateau after yielding, further indicating that the material undergoes continued plastic deformation during loading. When the strain rate is further increased to 5000 s^−1^, the recovered specimens exhibit a markedly higher degree of fragmentation. However, the relatively extended post-yield plateau in the corresponding stress–strain curves, together with the macroscopic deformation of the recovered specimens, suggests that fragmentation was preceded by substantial plastic deformation while the specimens sustained relatively high flow stresses. In addition, the observed fragmentation morphology may also be affected by subsequent interactions between the specimen and the SHPB system.

Comparison of the recovered specimens tested at the same strain rate but different temperatures reveals that temperature has a pronounced influence on the deformation and fracture morphologies. At 1000 s^−1^, the specimens tested at all temperatures retain good overall integrity without obvious fragmentation. With increasing temperature, both the axial shortening and radial expansion of the specimens become more pronounced, indicating a certain enhancement in the plastic deformation capability of the material at elevated temperatures. In contrast, at 5000 s^−1^, the effect of temperature on the degree of fragmentation is more significant. At 298 K, the specimens exhibit extensive fragmentation, with several relatively large fragments accompanied by numerous smaller fragments. When the temperature increases to 498 and 698 K, the degree of fragmentation is markedly reduced, with fewer small fragments being generated. The recovered morphologies are dominated by two relatively large fragments formed by inclined shear splitting. Overall, with increasing temperature, the specimens subjected to the lower strain rate exhibit more pronounced plastic upsetting, whereas those tested at the higher strain rate show reduced fragmentation. The observed failure morphology changes from extensive fragmentation with multiple pieces to a less fragmented inclined shear-splitting pattern.

**Figure 6:**
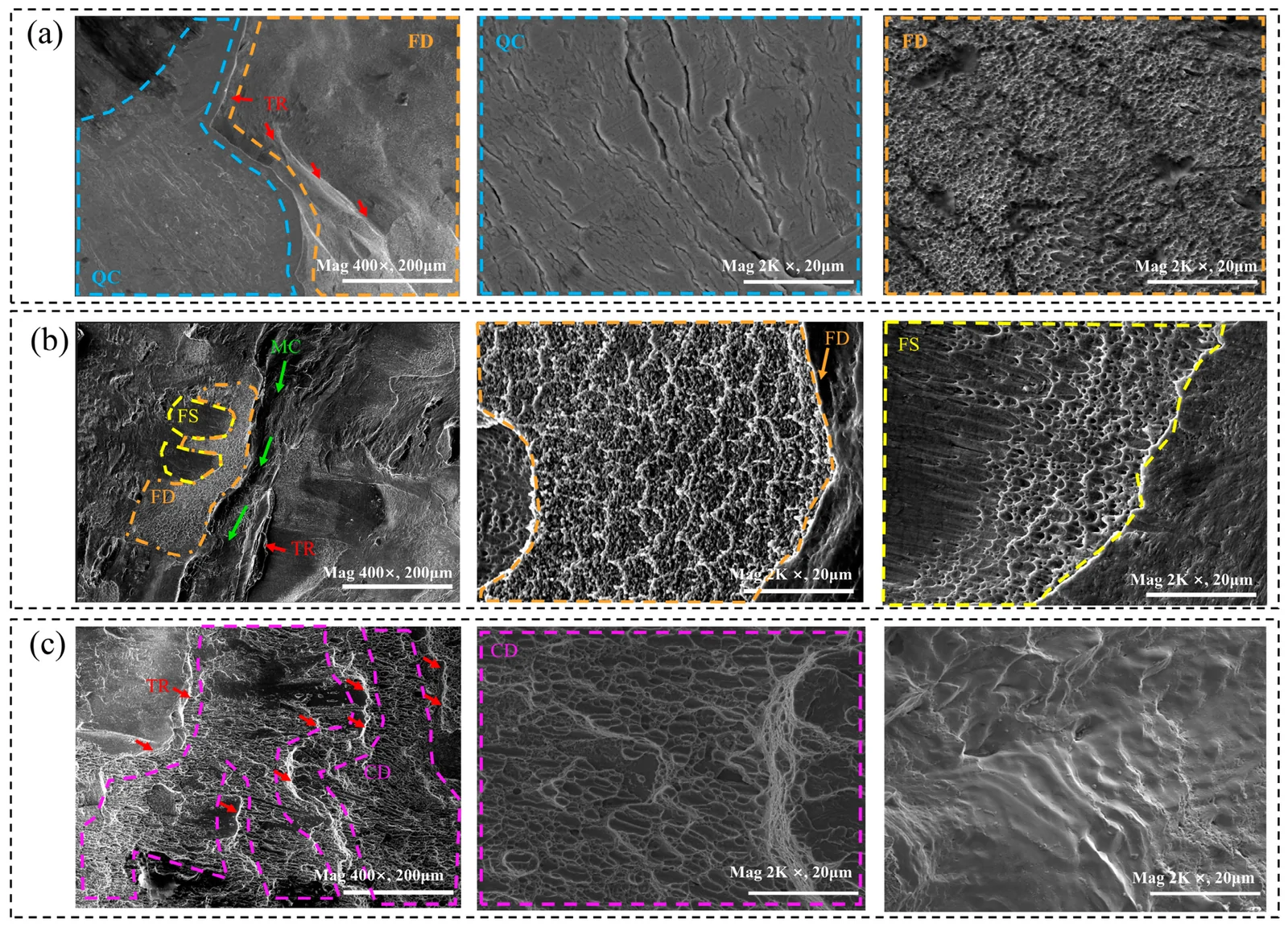
Typical fracture morphologies of specimens under different compression conditions: (**a**) quasi-static compression at 298 K and 0.00104 s^−1^; (**b**) dynamic compression at 298 K and 5000 s^−1^; (**c**) dynamic compression at 698 K and 5000 s^−1^. Note: MC, FS, QC, FD, CD, and TR denote main crack, fracture-step region, quasi-cleavage-like fracture region, fine-dimple region, coarse-dimple region, and tear-ridge-like features, respectively. The colored dashed regions and arrows are used to highlight the corresponding fracture features.

Figure 6a shows the typical fracture morphology of the specimen subjected to quasi-static compression at 298 K and 0.00104 s^−1^. The morphology was observed on the inclined shear crack surface formed during compression and exposed by subsequent manual separation, with the latter two images presenting higher-magnification views of selected local regions. At low magnification, distinct regional variations can be observed across the fracture surface. The fracture surface can be divided into two representative regions, including a relatively flat quasi-cleavage-like fracture region (QC) and a rougher fine-dimple region (FD). Tear-ridge-like features (TR), indicated by the red arrows, are observed near the boundary between these two regions. At higher magnification, the QC region exhibits relatively flat fracture features accompanied by several slender secondary cracks, which are consistent with quasi-cleavage-like fracture characteristics. In contrast, the FD region contains numerous fine and densely distributed dimples, indicating ductile fracture characteristics associated with microvoid coalescence.

Figure 6b shows the typical fracture morphology of the specimen subjected to dynamic compression at 298 K and 5000 s^−1^, with the latter two images presenting higher-magnification views of selected local regions. At low magnification, a main crack (MC) with a pronounced opening and a tortuous propagation path is observed in the central part of the fracture surface. A fracture-step region (FS) is visible adjacent to the main crack, while tear-ridge-like features (TR), indicated by the red arrows, are observed near the crack propagation region. The fracture surface surrounding the main crack also exhibits pronounced surface undulations. At higher magnification, the fine-dimple region (FD) contains numerous fine and densely distributed dimples, exhibiting ductile fracture characteristics associated with microvoid coalescence. The enlarged view of the fracture-step region (FS) reveals clear variations in local dimple morphology. Some areas contain elongated dimples with a preferred orientation, whereas the adjacent regions are dominated by fine equiaxed dimples.

Figure 6c shows the typical fracture morphology of the specimen subjected to dynamic compression at 698 K and 5000 s^−1^, with the latter two images presenting higher-magnification views of selected local regions. At low magnification, the fracture surface exhibits distinct morphological variations, including tear-ridge-like features (TR) and a coarse-dimple region (CD). The coarse-dimple region (CD) is relatively rough and contains abundant ductile fracture features. At higher magnification, the CD region is characterized by numerous coarse dimples, some of which are elongated with a preferred orientation, together with a certain number of finer dimples. These features exhibit ductile fracture characteristics associated with microvoid coalescence. In another enlarged region, the fracture surface is relatively continuous and shows pronounced surface undulations, together with numerous directionally distributed band-like and wavy features. Local irregular protrusions and depressions can also be observed, suggesting localized plastic-flow features.

## 4. Constitutive Model Calibration and Assessment

### 4.1. Calibration of Johnson–Cook Flow Stress Model Parameters

The preceding quasi-static and dynamic compression tests demonstrate that the medium-carbon Cr-Ni-Mo high-strength steel exhibits pronounced strain hardening, strain-rate strengthening, and thermal softening under different loading conditions. To quantitatively describe the dynamic compressive response of the material under the combined effects of plastic strain, strain rate, and temperature, and to provide material parameters for subsequent numerical simulations, the Johnson–Cook flow stress model was employed to characterize the plastic flow behavior of the material prior to failure under dynamic compression. The model is expressed as follows [27,28]:

(1) $$\sigma =\left[A+B\varepsilon^{n}\right]\left[1+C\ln\left(\frac{\dot{\varepsilon }}{{\dot{\varepsilon }}_{0}}\right)\right]\left[1-T^{*m}\right]$$

where *σ* is the equivalent flow stress, MPa; *ε* is the equivalent plastic strain; $\dot{\varepsilon }$ is the equivalent plastic strain rate, s^−1^; ${\dot{\varepsilon }}_{0}$ is the reference strain rate, taken as 0.00104 s^−1^; T* is the homologous temperature, defined as T* = (*T* − *T*_r_)/(*T_m_* − *T*_r_), where *T* is the test temperature, *T_r_* is the reference temperature, taken as 298 K, and *T_m_* is the melting temperature of the material, taken as 1748 K. *A* is the initial yield stress at the reference temperature and reference strain rate, MPa; *B* is the strain-hardening coefficient, MPa; *n* is the strain-hardening exponent; *C* is the strain-rate sensitivity coefficient; and *m* is the thermal-softening exponent.

**Figure 7:**
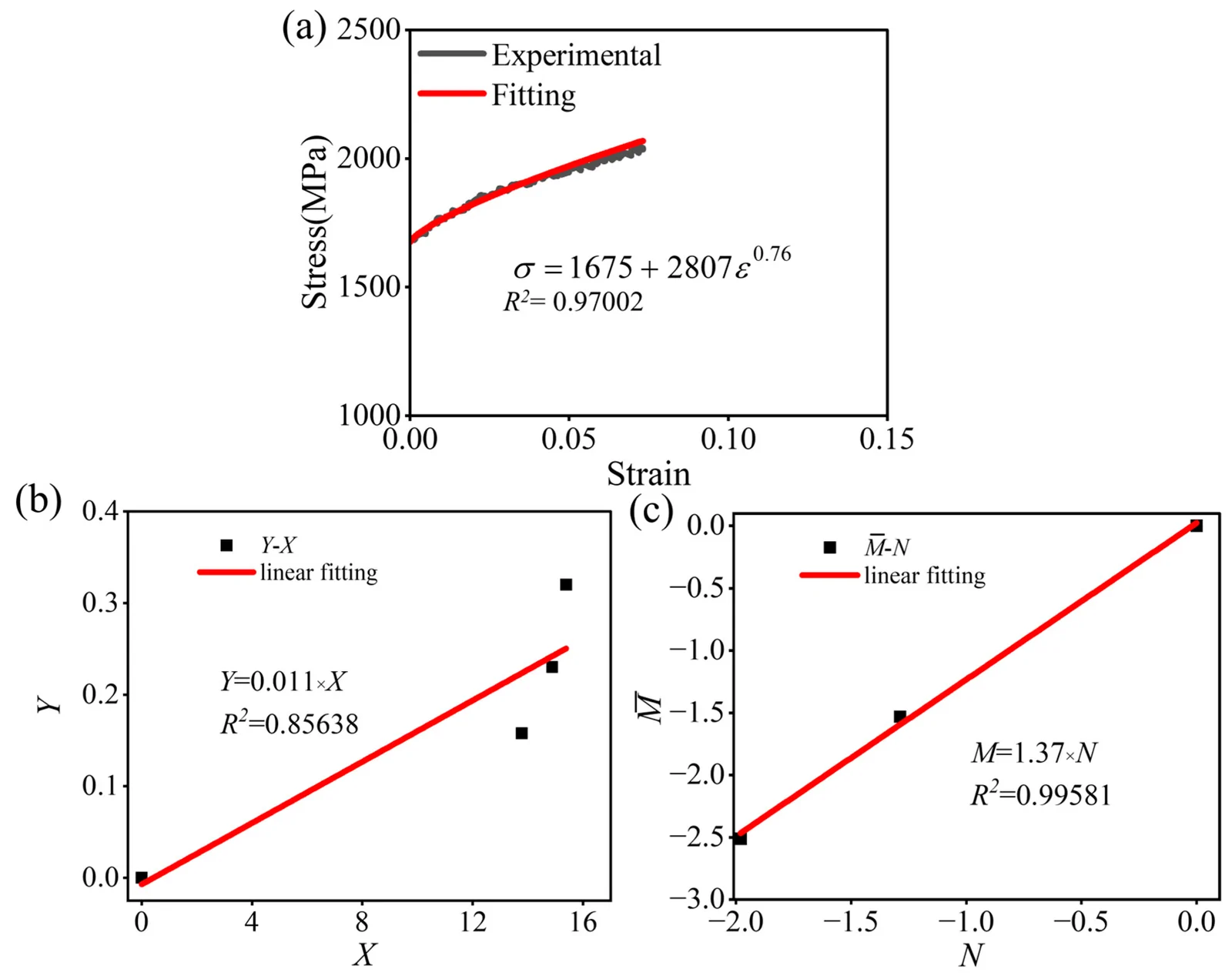
Calibration results of Johnson–Cook flow stress model parameters: (**a**) strain hardening parameters *B* and *n*; (**b**) strain-rate sensitivity coefficient *C*; (**c**) thermal softening exponent *m*.

A stepwise calibration procedure was adopted to determine the parameters of the Johnson–Cook model. First, under the reference temperature and reference strain-rate conditions, both the strain-rate and temperature terms reduce to unity, and the model is simplified to *σ* = (*A* + *Bε^n^*). The initial yield stress was determined from the quasi-static compression curve, giving *A* = 1675 MPa. The post-yield plastic region was then fitted, yielding *B* = 2807 MPa and *n* = 0.76, as shown in Figure 7a. Subsequently, the dynamic compression curves obtained at different strain rates at 298 K were used to determine the strain-rate sensitivity parameter. Data from the stable plastic-flow stage at each strain rate were used to establish a linear relationship between $Y=\sigma /(A+B\varepsilon_{p}^{n})-1$ and $X=\ln(\dot{\varepsilon }/{\dot{\varepsilon }}_{0})$. Linear fitting yielded *C* = 0.011, as shown in Figure 7b. Finally, the dynamic compression data obtained at different temperatures at a strain rate of 1000 s^−1^ were used to determine the thermal-softening parameter. Data from the stable plastic-flow stage at each temperature were used to establish a linear relationship between $M=\ln\left\{1-\sigma /\left\{\left(A+B\varepsilon^{n}\right)\left[1+C\ln(\dot{\varepsilon }/{\dot{\varepsilon }}_{0})\right]\right\}\right\}$ and *N* = lnT*. Linear fitting yielded *m* = 1.37, as shown in Figure 7c. The calibrated Johnson–Cook flow model parameters are summarized in Table 3.

### 4.2. Calibration of Johnson–Cook Damage Model Parameters

The Johnson–Cook damage parameters *D*_1_–*D*_4_ were identified through an inverse procedure combining SHPB experiments with numerical simulations. The experimental stress–strain responses obtained at 298 K under different strain rates, together with the post-test deformation and damage morphologies of the recovered specimens, served as the basis for parameter identification. *D*_1_–*D*_4_ were iteratively adjusted and selected based on a combined assessment of agreement with these responses and morphologies, while *D*_5_ remained uncalibrated. The Johnson–Cook damage model expresses the equivalent plastic strain at failure as follows:

(2) $$\varepsilon_{f}=\left[D_{1}+D_{2}e^{D_{3}\eta }\right]\left[1+D_{4}\ln\left(\frac{\dot{\varepsilon }}{{\dot{\varepsilon }}_{0}}\right)\right]\left[1+D_{5}T^{*}\right]$$

where *ε_f_* is the equivalent plastic strain at failure; *D*_1_–*D*_3_ describe the effect of stress triaxiality on the failure strain, *D*_4_ represents the strain-rate effect, and *D*_5_ represents the temperature effect; *η* is the stress triaxiality. In the present simulations, *D*_5_ was not calibrated in the present study because the damage-model identification and validation were performed at 298 K, corresponding to T* = 0, under which the temperature-dependent term does not affect the predicted failure strain.

The damage variable progressively accumulates with plastic deformation. The cumulative damage variable is defined as follows. When *D* = 1, the material is considered to have reached the failure criterion. In the numerical implementation, elements were deleted once the failure criterion was reached.

(3) $$D=\sum \frac{\Delta \varepsilon_{p}}{\varepsilon_{f}}$$

where *D* is the cumulative damage variable, and Δ*ε_p_* is the increment of equivalent plastic strain within the current calculation increment.

#### 4.2.1. SHPB Finite Element Model

The corresponding finite element model was established based on the SHPB dynamic compression tests and consisted of the incident bar, specimen, and transmitted bar. Both the incident and transmitted bars had a diameter of 20 mm and a length of 1500 mm, while the specimen dimensions were Ø5 mm × 5 mm. The pressure bars were modeled using an elastic material model with an elastic modulus of 2.10 × 10^5^ MPa and a density of 7.85 × 10^−9^ t/mm^3^. The specimen was modeled using the Johnson–Cook flow stress and damage models described above. The flow stress parameters were adopted from the calibration results presented in Section 4.1, while the initial values for inverse identification of the damage parameters were selected with reference to reported high-strength steels with similar chemical compositions and mechanical properties [29], namely *D*_1_ = 0.014, *D*_2_ = 0.015, *D*_3_ = 3.251, and *D*_4_ = 0.007. All components were discretized using solid elements. A mesh-sensitivity analysis was performed using specimen mesh sizes of 0.2, 0.4, and 0.6 mm at 298 K and 1000 s^−1^, with all other settings unchanged. Across the three meshes, the differences between the highest and lowest values were 7.1 MPa for maximum compressive stress and 0.00014 for maximum recorded strain. These variations were small relative to the corresponding response magnitudes, suggesting limited mesh sensitivity of these two quantities under the examined condition. The 0.2 mm specimen mesh was used consistently for parameter calibration and the subsequent simulations. The main regions of the pressure bars were meshed at 0.7 mm, with local refinement near the bar–specimen contact interfaces. Automatic surface-to-surface contact was defined between the incident bar and specimen and between the specimen and transmitted bar. The striker bar was not explicitly modeled. Instead, the experimentally measured incident stress-time history was applied as a uniformly distributed axial surface load to the outer end face of the incident bar and used as the loading input for the numerical simulations under the corresponding test conditions.

#### 4.2.2. Determination of Damage Parameters

**Figure 8:**
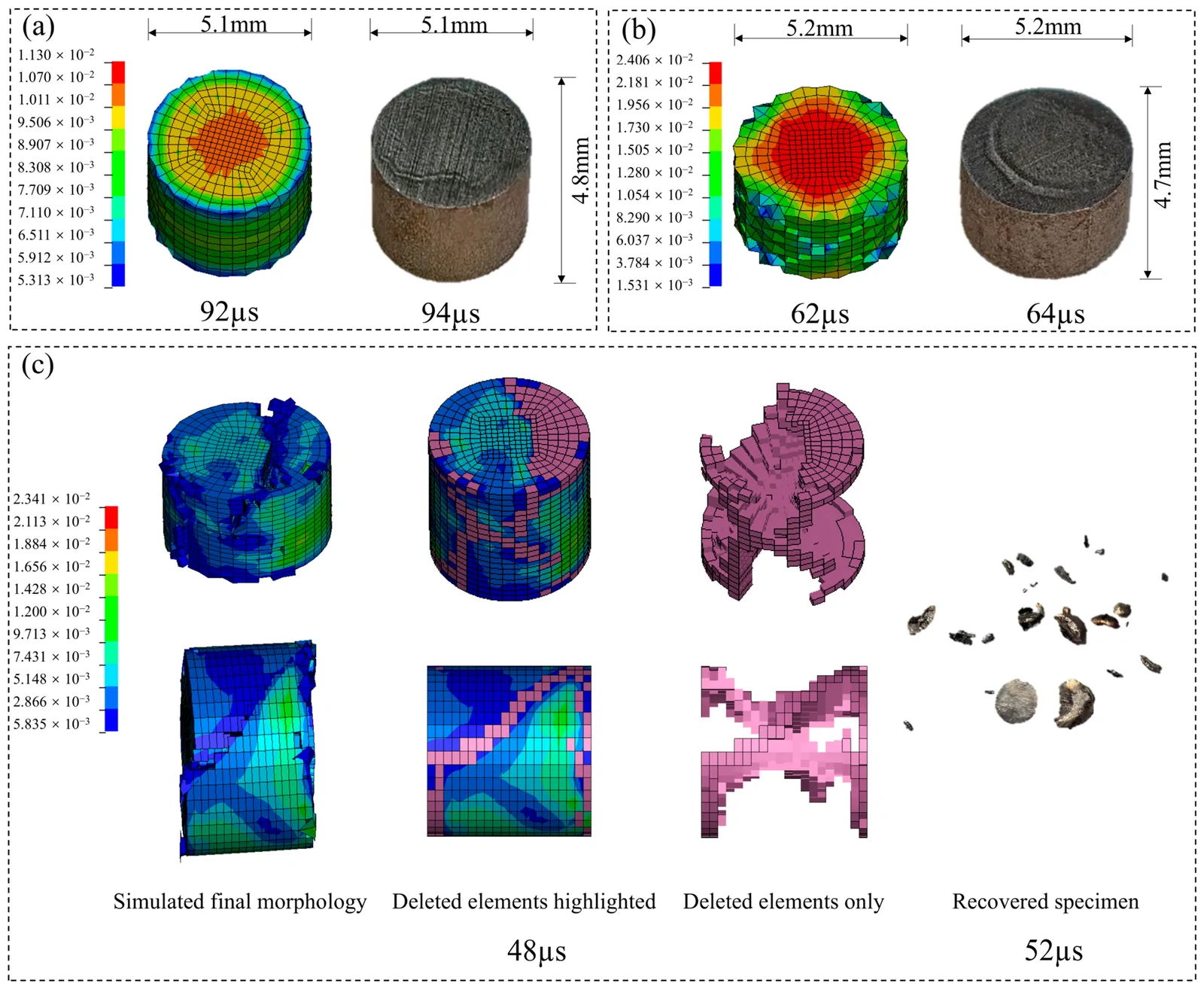
Comparison of the numerical deformation and damage characteristics with the experimentally recovered specimens at 298 K and different strain rates: (**a**) 1000 s^−1^; (**b**) 3000 s^−1^; (**c**) 5000 s^−1^. The color contours in the numerical results represent the von Mises stress. For the 5000 s^−1^ condition in (**c**), the deleted elements are additionally highlighted in magenta and displayed separately in the undeformed configuration to illustrate their spatial distribution. The magenta color is used only to identify deleted elements and does not correspond to the von Mises stress scale.

**Figure 9:**
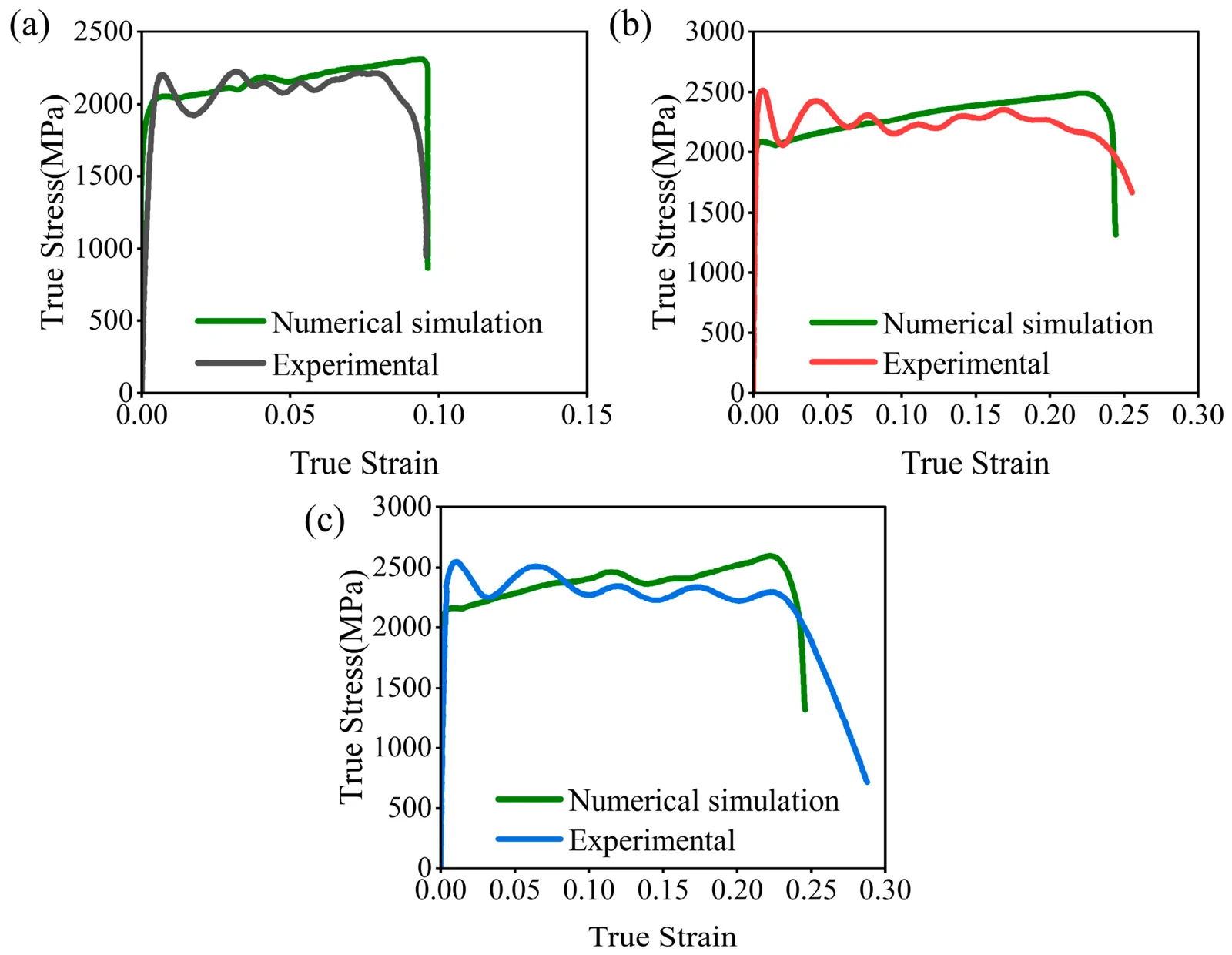
Comparison between representative experimental and numerical true stress–strain curves under different strain rates at 298 K: (**a**) 1000 s^−1^; (**b**) 3000 s^−1^; (**c**) 5000 s^−1^.

After multiple iterations of parameter adjustment, the Johnson–Cook damage parameters were identified. Figure 8 and Figure 9 present comparisons between the numerical predictions and experimental results in terms of specimen deformation and damage morphology, and stress–strain response, respectively.

Figure 8 compares the numerical deformation and damage characteristics with the morphologies of representative experimentally recovered specimens at 298 K and strain rates of 1000, 3000, and 5000 s^−1^. At 1000 s^−1^, the specimen in the numerical results mainly exhibited axial compression and radial expansion, with no obvious overall failure, which is generally consistent with the final deformation morphology of the experimentally recovered specimen. When the strain rate was increased to 3000 s^−1^, obvious inclined shear cracks appeared on the side surface of the specimen in the numerical results, and corresponding inclined shear cracks were also observed in the experimentally recovered specimen, showing good consistency in the main deformation and crack morphology. At 5000 s^−1^, the numerical specimen developed a pronounced inclined shear failure path and showed a tendency to separate into two major portions along the inclined shear crack, accompanied by localized element deletion in and around the shear-failure region. Figure 8c further presents the deleted-element distribution in the undeformed configuration, with the deleted elements highlighted in magenta. The deleted elements are mainly concentrated along the inclined failure region and several localized areas. The representative recovered specimen similarly exhibits pronounced inclined shear separation, with relatively large main fragments accompanied by smaller fragments and debris, which is generally consistent with the main failure characteristics reproduced by the numerical simulation. It should be noted that Figure 8c shows only one representative recovered specimen from those presented in Figure 5c. Because the final number and distribution of the recovered fragments may also be influenced by inertial separation, subsequent interactions, and the recovery process, the experimentally recovered fragments should not be interpreted as corresponding one-to-one with the deleted elements in the simulation. Overall, the numerical results reasonably reproduce the main characteristics of inclined shear failure, separation of the main body, and localized fragmentation under this condition.

Figure 9 compares the representative experimental and numerically simulated true stress–strain curves under different strain rates at 298 K. Overall, the numerical curves capture the main features of the experimental stress response under all three strain-rate conditions. The quantitative comparison of the maximum compressive stress and maximum recorded strain between the simulations and the representative experimental curves is summarized in Table 4.

As shown in Table 4, the simulated maximum compressive stresses agree well with the experimental results, with relative errors below 3.6% under all investigated strain-rate conditions. The predicted maximum recorded strains also show reasonable agreement with the experimental values, although a larger deviation is observed at 5000 s^−1^, where the simulation underestimates the experimental strain by 14.60%. This discrepancy indicates the limitation of the current model in reproducing the extent of deformation associated with damage evolution under high strain-rate loading. Nevertheless, the model reasonably captures the overall stress response and deformation characteristics within the investigated conditions.

The identified Johnson–Cook damage model parameters are summarized in Table 5. Overall, the identified Johnson–Cook model captures the main features of the stress response and the observed deformation and damage morphologies under the investigated compression conditions at 298 K. This parameter set, although not necessarily unique, provides a description of the experimental responses used for parameter identification within the investigated compression conditions. Because *D*_5_ was not calibrated, the present damage model does not explicitly describe the temperature dependence of the failure strain. Nevertheless, within the present formulation, temperature can affect fragmentation indirectly through the thermal-softening effect on plastic flow, which may permit greater plastic deformation before the local failure criterion is reached, rather than through a direct temperature dependence of the failure strain *ε_f_*. This may partly account for the reduced fragmentation observed at elevated temperatures. Therefore, the present validation of the damage model is limited to the investigated strain-rate conditions at 298 K, and its extension to other temperatures and stress states requires further calibration and independent validation.

## 5. Discussion

### 5.1. Effects of Strain Rate and Temperature on Microscopic Fracture Characteristics

Under different loading conditions, the fracture surfaces of the medium-carbon Cr-Ni-Mo high-strength steel exhibited mixed ductile and quasi-cleavage-like fracture characteristics to varying degrees, and variations in strain rate and temperature markedly altered the microscopic fracture morphology. Under quasi-static compression, relatively flat quasi-cleavage-like fracture regions coexisted with fine equiaxed dimple regions, indicating the coexistence of quasi-cleavage-like features and ductile dimple fracture. When the strain rate was increased to 5000 s^−1^, numerous fine dimples were still present on the fracture surfaces, together with oriented elongated dimples that were not evident under quasi-static conditions, accompanied by pronounced fracture steps and tear ridges. These features suggest that the material still exhibited pronounced plastic deformation at high strain rates, while localized shear became more prominent, consistent with the macroscopic inclined shear cracking and fragmentation observed under high-strain-rate conditions. Similar coexistence of dimpled fracture features and macroscopic shear failure has been reported for BQ&P steel [30] and quenched-and-tempered AISI 4340 steel [31], while pronounced localized shear and adiabatic shear band evolution have also been observed in high-strength steels under high-strain-rate loading [17].

On the other hand, when the strain rate was maintained at 5000 s^−1^ and the temperature was increased to 698 K, coarser dimples became more prominent on the fracture surfaces, and relatively continuous band-like and wavy surface features appeared, suggesting features associated with enhanced plastic deformation and ductile fracture at elevated temperature. These changes were consistent with the macroscopic trend of reduced fragmentation. The accompanying decrease in compressive stress with increasing temperature is consistent with the thermal softening reported for 34CrNi3MoA steel [16]. Overall, within the investigated conditions, increasing strain rate promoted more prominent shear-related fracture features, whereas increasing temperature tended to promote plastic deformation and ductile fracture features [32].

### 5.2. Numerical Simulation of Deformation and Damage Behavior Under Dynamic Compression

**Figure 10:**
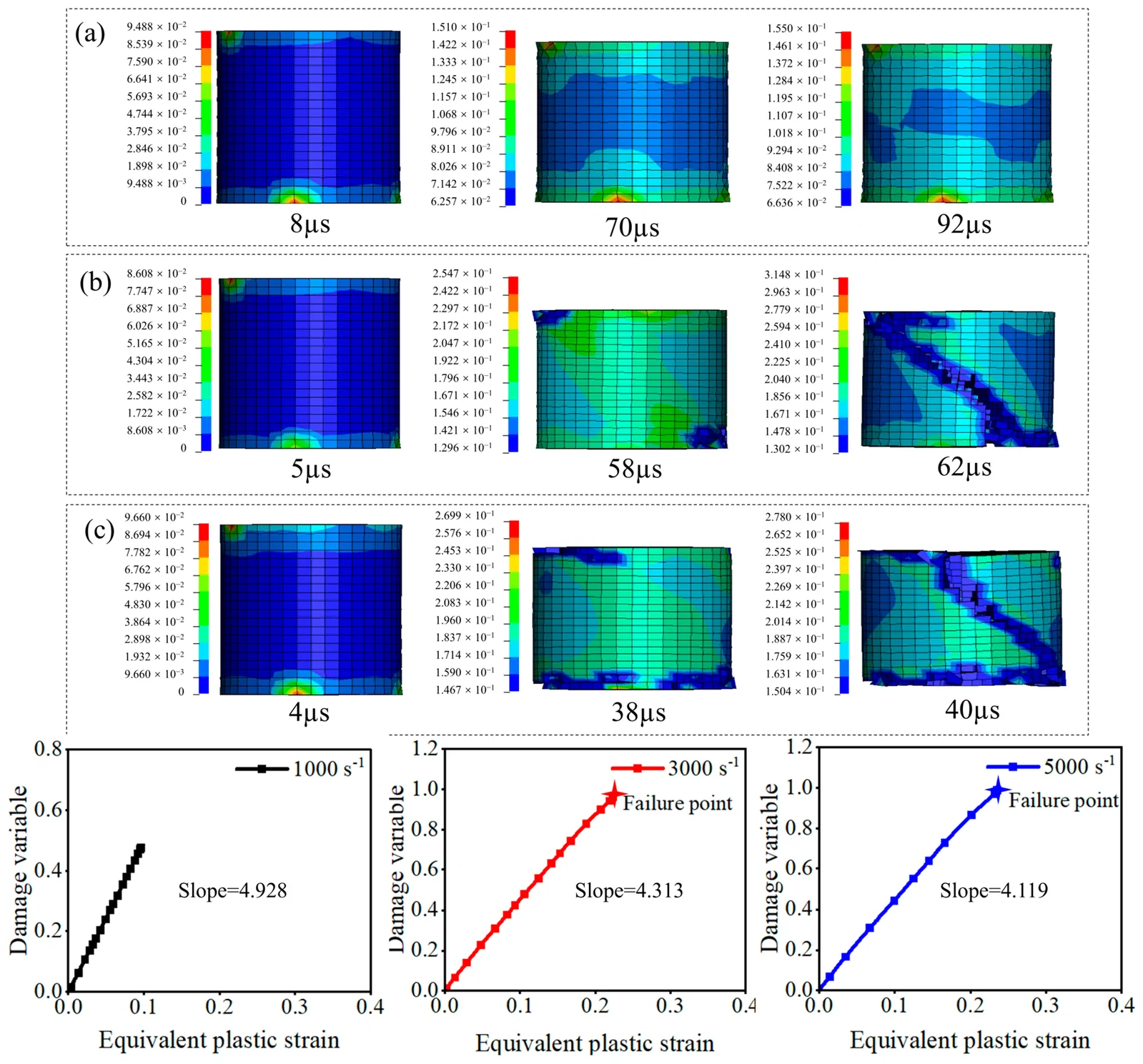
Equivalent plastic strain distribution and damage variable evolution at different strain rates: (**a**) 1000 s^−1^; (**b**) 3000 s^−1^; (**c**) 5000 s^−1^.

From the equivalent plastic strain contours in Figure 10, it can be seen that the plastic deformation and damage evolution of the specimens differ markedly under different strain rates. At 1000 s^−1^, the equivalent plastic strain within the specimen was generally relatively continuous and uniform; as loading proceeded, the specimen mainly exhibited axial compression and radial expansion, and still maintained good overall integrity at the end of loading, without obvious localized damage or failure. When the strain rate was increased to 3000 s^−1^, the specimen underwent considerable plastic deformation prior to damage localization, and the heterogeneity of the equivalent plastic strain distribution gradually increased, with an inclined region of localized plastic strain appearing; in the later stage of loading, localized damage developed along this region. Under the condition of 5000 s^−1^, the specimen likewise underwent considerable plastic deformation prior to damage localization, and the inclined region of localized plastic strain appeared earlier, followed by the formation of shear cracks within this region. Overall, with increasing strain rate, the specimens exhibit greater plastic deformation during the simulated loading process, while the heterogeneity of the equivalent plastic strain distribution gradually increases, and the inclined region of localized plastic strain and shear cracks appear earlier, indicating a greater tendency toward localized shear deformation and damage at higher strain rates under the investigated loading conditions.

Further, from the relationship between the damage variable *D* and the equivalent plastic strain in Figure 10, it can be seen that under all three strain rates, the damage variable increased approximately linearly with increasing equivalent plastic strain, indicating that material damage continuously accumulated with plastic deformation. At 1000 s^−1^, *D* was approximately 0.48 at the end of loading; the specimen mainly underwent overall plastic compression, without reaching the failure criterion. When the strain rate was increased to 3000 and 5000 s^−1^, the damage variable eventually reached *D* = 1 in both cases, corresponding to the predicted failure of the specimens and the experimentally observed inclined shear cracks. The fitting slopes of the *D*-equivalent plastic strain relationship under the three strain rates were 4.928, 4.313, and 4.119, respectively. According to the Johnson–Cook damage formulation, these slopes are associated with the inverse of the failure strain rather than representing independent damage coefficients. Therefore, the decrease in the fitted slope with increasing strain rate can be interpreted as an increase in the effective failure strain, corresponding to greater plastic deformation before failure.

## 6. Conclusions

(1) The mechanical property test results indicate that the medium-carbon Cr-Ni-Mo high-strength steel exhibits pronounced strain-rate strengthening and thermal softening characteristics. At 298 K, the mean quasi-static tensile peak stress is 2113.3 MPa, while the mean maximum compressive stress is 1988.1 MPa. When the strain rate is increased to 5000 s^−1^, the mean maximum compressive stress reaches 2500.9 MPa, and the strain range corresponding to the plastic flow stage is markedly enlarged. Taking 1000 s^−1^ as an example, as the temperature increases from 298 to 698 K, the mean maximum compressive stress decreases from 2263.1 to 1722.2 MPa, corresponding to a reduction of approximately 23.9%; however, the strain range corresponding to the plastic flow stage also increases to some extent.

(2) The Johnson–Cook model parameter identification results indicate that the established model can reasonably describe the main features of the experimental responses. The flow stress model parameters were determined as *A* = 1675 MPa, *B* = 2807 MPa, *n* = 0.76, *C* = 0.011, and *m* = 1.37; the damage parameters were determined as *D*_1_ = 0.11, *D*_2_ = 0.05, *D*_3_ = 1.6, *D*_4_ = 0.008, while *D*_5_ was not calibrated in the present study. Over the strain rate range of 1000–5000 s^−1^ at 298 K, the relative errors between the numerically simulated maximum compressive stresses and the corresponding representative experimental results were all less than 4%. However, the maximum recorded strain at 5000 s^−1^ was underestimated by 14.60%, indicating a limitation in reproducing the extent of deformation under this condition. Meanwhile, the main morphological characteristics of the specimens, transitioning from axial compression and radial expansion at lower strain rates to inclined cracking, localized damage, and partial fragmentation at higher strain rates, were qualitatively reproduced.

(3) Macroscopic and microscopic morphology observations indicate that the deformation and fracture characteristics of the medium-carbon Cr-Ni-Mo high-strength steel are significantly affected by strain rate and temperature. As the strain rate increases, the specimens, initially dominated by plastic compressive deformation at low strain rates, gradually exhibit inclined cracking, and pronounced fragmentation was observed in the recovered specimens at 5000 s^−1^. As the temperature increases, the degree of fragmentation of the specimens decreases at 5000 s^−1^. At the microscopic scale, the fracture surfaces under different loading conditions all exhibit ductile fracture characteristics to varying degrees. Under quasi-static conditions, quasi-cleavage-like regions and fine equiaxed dimples are observed. When the strain rate is increased to 5000 s^−1^, pronounced oriented elongated dimples, fracture steps, and tear ridges appear. Under the conditions of 5000 s^−1^ and 698 K, coarser dimples and features associated with plastic deformation become more prominent. Overall, increasing strain rate mainly promotes localized shear deformation, whereas increasing temperature makes the plastic deformation and ductile fracture characteristics more pronounced.

(4) The numerical simulation results indicate that, with increasing strain rate, the equivalent plastic strain during dynamic compression gradually localizes, forming an inclined region of localized plastic strain, followed by inclined shear cracking. At 1000 s^−1^, the specimen was dominated by axial compression and radial expansion, and the damage variable *D* was approximately 0.48 at the end of loading, without reaching the failure criterion. When the strain rate was increased to 3000 and 5000 s^−1^, the inclined region of localized plastic strain and shear cracks appeared earlier and became more pronounced, and the damage variable eventually reached *D* = 1. The fitting slopes of the *D*-equivalent plastic strain relationship under the three strain rates were 4.928, 4.313, and 4.119, respectively, indicating that, in the present simulations, increasing strain rate was associated with a lower slope of the *D*-equivalent plastic strain relationship.

## Figures and Tables

**Table 1:** Chemical composition of the medium-carbon Cr-Ni-Mo high-strength steel.

Element	C	Si	Mn	Cr	Ni	Mo	P	S	Fe
wt.%	0.45	1.75	0.55	0.9	3.85	0.65	0.0115	0.0125	Bal.

Note: The chemical composition values were provided by the material supplier. No independent compositional analysis was performed in the present study.

**Table 2:** Summary of repeated SHPB test results at different temperatures and strain rates.

Nominal Strain Rate (s^−1^)	n	Temperature (K)	Measured Strain Rate (s^−1^)	Maximum Compressive Stress (MPa)	Maximum Recorded Strain
1000	3	298	994.52	2263.1 ± 45.9	0.092 ± 0.004
		498	1004.13	2036.7 ± 47.2	0.108 ± 0.003
		698	1000.77	1722.2 ± 34.6	0.113 ± 0.006
3000	3	298	2994.14	2451.3 ± 57.0	0.253 ± 0.003
		498	2987.49	2130.5 ± 66.9	0.264 ± 0.008
		698	3016.98	1909.1 ± 14.6	0.275 ± 0.008
5000	3	298	4987.59	2500.9 ± 76.7	0.281 ± 0.007
		498	5014.83	2222.6 ± 44.4	0.302 ± 0.005
		698	4990.05	2077.5 ± 63.8	0.333 ± 0.008

Note: Maximum compressive stress and maximum recorded strain are reported as mean ± standard deviation. The measured strain rate for each test was obtained by averaging the strain-rate history over the relatively stable loading stage.

**Table 3:** Johnson–Cook flow stress model parameters of the medium-carbon Cr-Ni-Mo high-strength steel.

Parameter	*A* (MPa)	*B* (MPa)	*n* (–)	*C* (–)	*m* (–)	${\dot{\varepsilon }}_{0}$ (s^−1^)	*T_r_* (K)	*T_m_* (K)
Value	1675	2807	0.76	0.011	1.37	0.00104	298	1748

**Table 4:** Comparison between representative experimental and numerical results at different strain rates.

Strain Rate (s^−1^)	Maximum Compressive Stress (MPa)		Relative Error (%)	Maximum Recorded Strain		**Relative** Error (%)
	Experiment	Simulation		Experiment	Simulation	
1000	2226.1	2305.9	3.59	0.09569	0.09629	0.63
3000	2498.4	2475.0	0.94	0.25537	0.24463	4.21
5000	2546.5	2564.7	0.71	0.28788	0.24586	14.6

Note: The experimental values correspond to the representative stress–strain curves shown in Figure 9.

**Table 5:** Initial and identified Johnson–Cook damage model parameters for the medium-carbon Cr-Ni-Mo high-strength steel.

Parameter	Initial Value	Final Value	Method
*D*_1_ (–)	0.014	0.11	Inverse calibration
*D*_2_ (–)	0.015	0.05	Inverse calibration
*D*_3_ (–)	3.251	1.6	Inverse calibration
*D*_4_ (–)	0.007	0.008	Inverse calibration
*D*_5_ (–)	–	–	Not calibrated

Note: Initial values of *D*_1_–*D*_4_ were selected with reference to [29]. *D*_1_–*D*_4_ were identified and assessed at 298 K. *D*_5_ was not calibrated in the present study because the damage-model identification and validation were limited to 298 K, where T* = 0. Extension to other stress states and temperatures requires further calibration and independent validation.

## Data Availability

The original contributions presented in this study are included in the article. Further inquiries can be directed to the corresponding author.
